# Impact of rehabilitation services on employment outcomes for individuals with physical disabilities: a propensity score matching analysis

**DOI:** 10.1186/s12889-024-19015-6

**Published:** 2024-06-07

**Authors:** Han Nah Park, Su Jin Lee, Ju Young Yoon

**Affiliations:** 1 College of Nursing, Seoul National University, 103 Daehak-ro, Jongno-gu, Seoul, 03080 South Korea; 2 Research Institute of Nursing Science, Seoul National University, Seoul, Republic of Korea; 3 Department of Nursing, Kyungdong University, Wonju, Republic of Korea

**Keywords:** Persons with disabilities, Rehabilitation, Employment, Propensity score

## Abstract

**Background:**

Choosing a suitable job and leading a fulfilling professional life is vital for individuals, regardless of disability. Governments provide rehabilitation services to promote employment for individuals with disabilities, but research on their effects is limited. This study aimed to examine the impact of rehabilitation services on employment among people with physical disabilities in South Korea using propensity score matching.

**Methods:**

This study utilized an observational research design. Data were obtained from the 2020 National Survey of Disabled Persons, including 1,757 individuals aged 20 or older with physical disabilities. Descriptive statistics, chi-square and independent t-tests, logistic regression, and propensity score matching were employed.

**Results:**

The results for employment of individuals with physical disabilities showed no difference between the with rehabilitation services and the without rehabilitation services group. Based on subgroup analysis, when individuals with physical disabilities who rated their subjective health status low received rehabilitation services, it had a positive effect on employment.

**Conclusions:**

The results of this study could serve as foundational data for future policies and educational directions concerning rehabilitation services for persons with disabilities.

**Supplementary Information:**

The online version contains supplementary material available at 10.1186/s12889-024-19015-6.

## Background

An essential aspect of the human experience regardless of disability status is having a job that aligns with one’s abilities and offers satisfaction and facilitates a fulfilling professional life [1]. Particularly for individuals with disabilities, employment not only serves as a means of livelihood but also enhances their quality of life and personal dignity by fostering a stable lifestyle. Employment also promotes social integration by narrowing the social, economic, and psychological gaps between individuals with and without disabilities [2].

However, people with disabilities face constraints in their occupational choices due to their physical and mental limitations. They often encounter difficulties securing employment in equitable competition with individuals without disabilities because of social discrimination and prejudice. According to a survey on the economic activity status of persons with disabilities in 2020, 801,039 (31.3%) of the 2,557,895 registered individuals with disabilities aged 15 years or older participated in economic activities, a stark contrast to the 62.7% participation rate among persons without disabilities. In addition, the employment rate for individuals with disabilities was 29.5%, compared to 60.4% for persons without disabilities, further highlighting the challenges individuals with disabilities face in obtaining employment. In South Korea, there are 15 recognized types of disabilities: physical, brain lesion, visual, hearing, speech, facial, kidney, heart, liver, respiratory, intestinal leukemia, epilepsy, intellectual, autistic, and mental disorders. Among those who are employed, the employment rate of those with facial disabilities is 48.1%, followed by those with liver disabilities at 41.3%, those with visual impairment at 38.5%, and those with physical disabilities at 36.8%. Given that physical disabilities account for the largest proportion of disabilities, and people with physical disabilities have the fourth highest employment rate among the registered disability types, the findings obtained from studying individuals with physical disabilities could be extrapolated to all disability types and have scalability [3]. Thus, the results need to be closely analyzed.

One reason individuals with disabilities face challenges securing employment is companies’ reluctance to hire them due to the higher costs associated with human capital development and the installation of functional aids. To address this issue, the government and local communities provide various services to individuals with disabilities, including rehabilitation services [4]. These rehabilitation services aim to help individuals overcome their disabilities and reintegrate into society by offering tailored and comprehensive support through medical, professional, educational, and social-psychological interventions from the onset of the physical disability [5]., Rosenfield [6] demonstrated that rehabilitation services had a greater impact on life satisfaction than psychiatric treatment for chronic mental patients, and Aulmann [7] reported a reduction in labor loss levels from 32.9 to 20.5%. These findings indicate that providing appropriate rehabilitation services to individuals with disabilities has a positive effect not only on their satisfaction but also on their reintegration into society, underscoring the effectiveness of such services. As a result, rehabilitation services play a significant role in influencing the employment of individuals with disabilities. This study examines the impact of rehabilitation services on employment among people with physical disabilities and presents foundational data to establish effective employment policies for persons with disabilities.

### Aims

The primary aim of this study is to examine the impact of utilizing rehabilitation services on the employment of individuals with physical disabilities using propensity score matching. The secondary aim is to identify how the characteristics of each subgroup affect the employment of individuals with physical disabilities using subgroup analysis.

## Methods

### Data and participants

This cross-sectional study analyzed data from the 2020 National Survey of Disabled Persons conducted by the Ministry of Health and Welfare and the Korea Institute for Health and Social Affairs. This is a nationally representative survey of community-dwelling people with disabilities in South Korea conducted every three years. The National Survey of Disabled Persons data are available on the health and welfare data portal website (https://data.kihasa.re.kr/kihasa/kor/contents/ContentsList.html). In 2020, 7,025 registered individuals with disabilities participated in the survey without considering household sampling due to the 2019 pandemic [3].

The inclusion criterion for the current study was individuals with physical disabilities aged $\ge$20 years. All individuals with missing values in their survey were excluded because propensity score matching should not include missing values. A total of 1,757 eligible participants were extracted from a dataset of 7,025 samples (Appendix A).

### Measures

#### Dependent variable

The question for employment was “Did you work for more than an hour for income last week?” with two possible responses: “yes” and “no.” If the answer was “yes,” it was defined as being employed.

#### Independent variable

Based on previous studies, the independent variables were rehabilitation services [8–10], age [8, 9, 11, 12], gender [8–11, 13], spouse [8–10, 12, 14], education [8, 10–12], monthly household income [11, 14], degree of disability [8, 9, 11, 13, 14], and subjective health status [11, 14]. The question for rehabilitation services was, “Is there any rehabilitation you are currently undergoing?” and was recorded as a binary value, with “yes” responses classified as “used rehabilitation services (= 1)” and “no” responses as “did not use rehabilitation services (= 0).” The question for age, “What is your age?” was recorded as a continuous variable. The question for gender was, “What is your gender?” This variable was also recorded as a binary value with two responses, “man (= 1)” and “woman (= 0).” The answer to the marital status question, “Are you married?” was recoded as a binary variable. A response of “yes” was classified as “has spouse (= 1),” and “widowed, divorced, separated, or never married” as “no spouse (= 0).” The question for education was, “What is your highest level of education?” and was recorded as follows: “pre-school, no school, elementary school (= 0)”; “elementary or under elementary graduation (= 1)”; “middle school” and “middle school graduation (= 2)”; “high school,” recorded as “high school graduation (= 3)”; “college, university, over graduation school,” recorded as “over college (= 4).” The question for monthly household income, “What was the average monthly household income during 2019 (2019.01.01 to 2019.12.31)?” was recorded as a continuous variable from 0 Korean won. The question for the degree of disability was, “What is the registered degree of disability?” This was a binary variable, and the answers were defined as “severe (= 1)” and “mild (= 0).” Subjective health status was assessed using a single question—“How do you feel about your health in general?”—on a 5-point Likert scale from “very bad (= 0)” to “very good (= 5).” A higher score indicated a better subjective health status.

### Statistical analysis

Data analysis was conducted using SPSS Statistics 23.0 (IBM Corp., Armonk, NY, USA) and SAS 9.4. First, we conducted propensity score matching to reduce selection bias between the group with rehabilitation services and the group without rehabilitation services. In this study, the nearest-neighbor matching method and caliper matching method were combined and applied. This study applied 1:1 ratio matching, and the caliper range was 0.01. To verify the results of propensity score matching, a paired t-test was conducted and the standardized mean difference was determined [15]. In the propensity score matching analysis, the dependent variable was set as the use of rehabilitation services. Based on previous studies, the independent variables were gender [16, 17], age [16, 18], monthly household income [16, 18], degree of disability [16], and disability origin [17]. Appendix B provides further details about the description of the variables used in propensity score matching. Second, the chi-square test and independent t-test were conducted to check whether there was a difference in employment according to general characteristics in the propensity score-matched data. Finally, logistic regression analysis was performed to estimate the effect of rehabilitation services on the employment of individuals with physical disabilities. Furthermore, factors influencing the employment of individuals with disabilities, such as gender (male, female), degree of disability (mild, severe), and subjective health status (good, bad), were divided into subgroups. Subgroup analysis was then conducted to determine whether the characteristics of each subgroup affected employment. Sandwich estimators were applied to the logistic regression analysis.

## Results

### Propensity score matching

As a result of propensity score matching, 478 people were matched in both the with rehabilitation services group and the without rehabilitation services group. Before propensity score matching, there were differences in all variables except for those aged 50–59, 60–69, monthly household income of between 1,000,000 and 2,000,000 Korean won, and the origin of the disability: innate or acquired disability. However, after propensity score matching, no variables were found to be statistically significant, indicating that the distribution was similar for all variables constructed after matching in the with rehabilitation services group and without rehabilitation services group (Table 1).

**Table 1:** Analysis of the difference before and after propensity score matching

								(UNIT: *N*(%))	
		Before matching (*N* = 1,757)				After matching (*N* = 956)			
		Rehabilitation services		t	p	Rehabilitation services		t	p
		Yes (*n* = 499)	No (*n* = 1,258)			Yes (*n* = 478)	No (*n* = 478)		
Gender	Men	230 (44.7)	785 (60.7)	-5.777	< 0.001 ***	223 (45.3)	224 (44.9)	-0.064	0.949
	Women	269 (55.3)	473 (39.3)	5.777	< 0.001 ***	255 (54.7)	254 (55.1)	0.064	0.949
Age (years)	20–39	11 (2.6)	44 (4.3)	-2.413	0.016*	7 (1.8)	7 (1.5)	0.000	1.000
	40–49	25 (5.3)	131 (10.4)	-4.737	< 0.001 ***	24 (5.3)	24 (5.5)	0.000	1.000
	50–59	94 (18.3)	263 (21.7)	-1.711	0.088	90 (18.3)	91 (19.4)	-0.084	0.933
	60–69	141 (28.4)	347 (27.0)	0.707	0.480	138 (29.3)	135 (28.6)	0.219	0.827
	70–79	145 (26.7)	305 (22.5)	3.005	0.003 **	138 (26.1)	136 (25.4)	0.146	0.884
	Over 80	83 (18.7)	168 (14.1)	2.757	0.006 **	81 (19.1)	85 (19.6)	-0.351	0.726
Monthly household income (10,000 KRW)	< 100	189 (36.8)	373 (30.4)	4.663	< 0.001 ***	185 (38.0)	189 (40.1)	-0.268	0.789
	100–<200	141 (27.1)	344 (25.3)	0.718	0.473	133 (26.6)	130 (24.9)	0.220	0.826
	200–<300	77 (17.4)	226 (18.3)	-2.041	0.042*	71 (16.6)	73 (16.0)	-0.179	0.858
	300–<400	41 (8.8)	144 (12.1)	-2.140	0.033*	41 (9.3)	39 (8.4)	0.239	0.811
	≥400	51 (9.8)	171 (13.8)	-2.862	0.004 **	48 (9.6)	47 (10.6)	0.108	0.914
Degree of disability	Mild (grade 4–6)	359 (84.2)	786 (78.8)	4.674	< 0.001***	344 (84.4)	345 (85.4)	-0.071	0.944
	Severe (grade1–3)	140 (15.8)	472 (21.2)	-4.674	< 0.001***	134 (15.6)	133 (14.6)	0.071	0.944
Disability origin	Congenital	184 (39.1)	455 (34.8)	0.451	0.653	175 (38.9)	181 (39.1)	-0.408	0.684
	Acquired	278 (52.7)	727 (58.4)	-1.355	0.176	273 (54.1)	270 (55.2)	0.199	0.842
	Unknown	37 (8.2)	76 (6.8)	1.993	0.047*	30 (7.0)	27 (5.7)	0.437	0.662

**Fig. 1:**
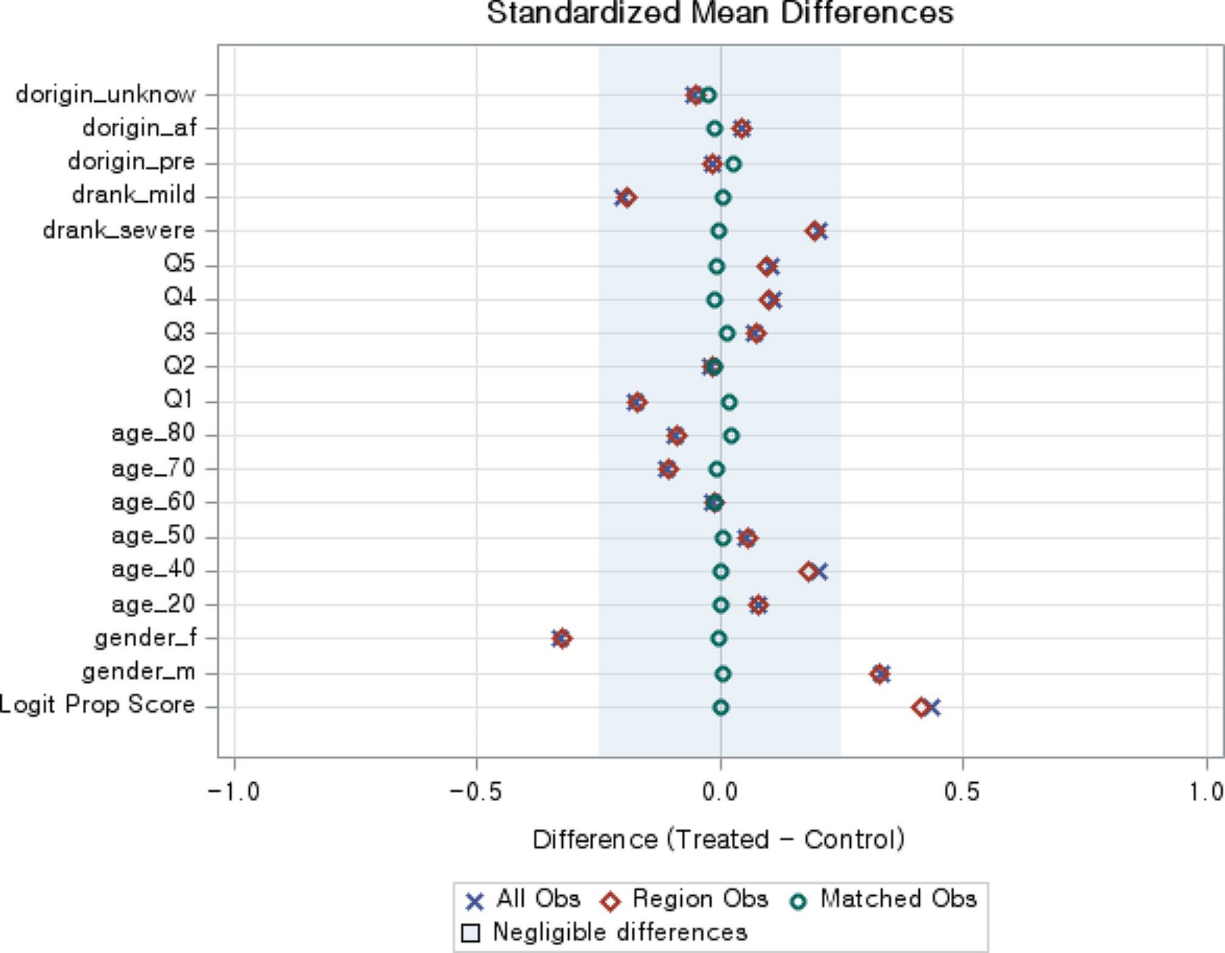
Standardized mean differences. Before and after propensity score matching, the variables of the standardized mean difference. The variables used for matching were gender, age, monthly household income, degree of disability, and disability origin. The red diamond is the standardized mean difference of the variable before matching, and the green circle is the standardized mean difference of the variable after matching

**Fig. 2:**
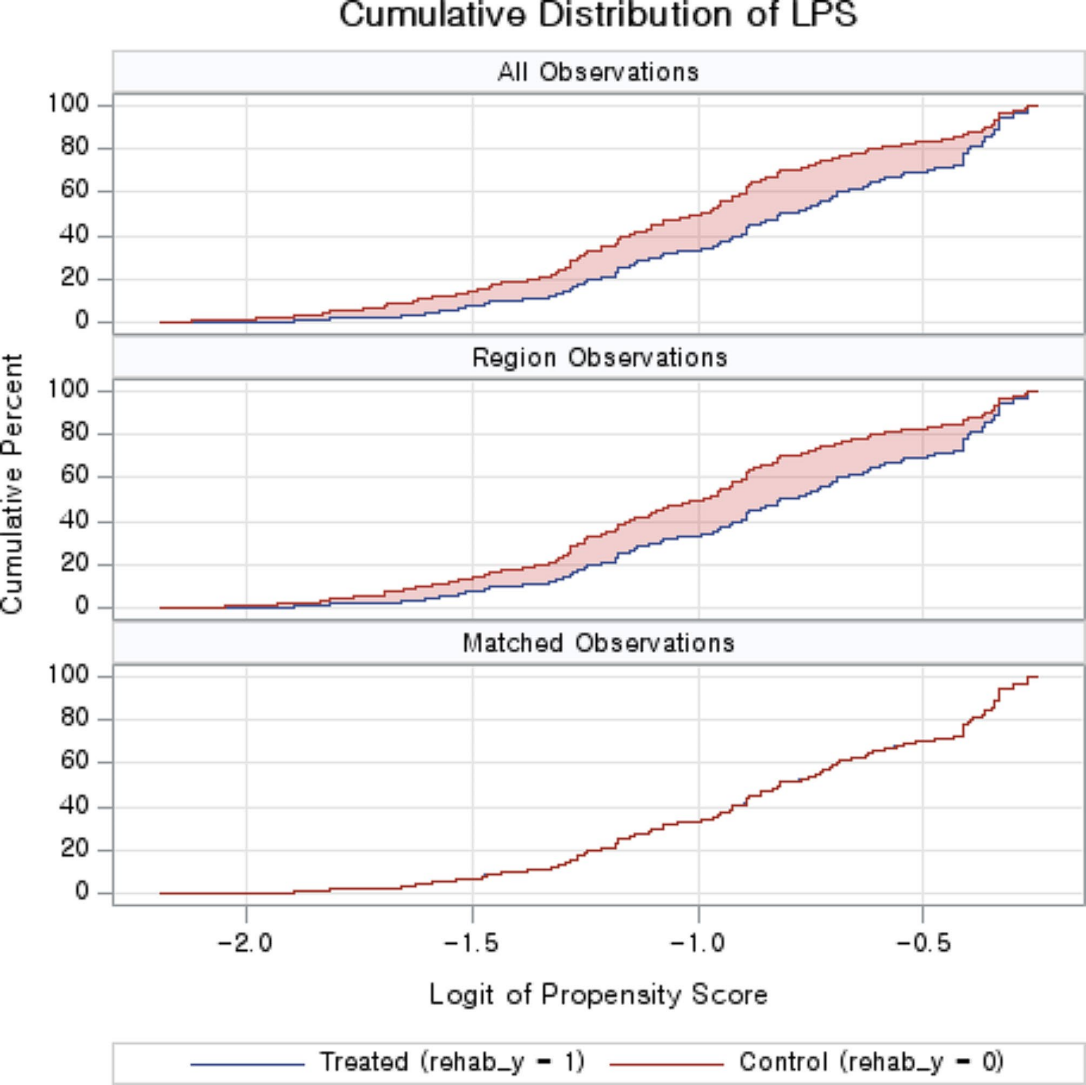
Cumulative distribution of logit of propensity score. The cumulative distribution of logit of propensity score between the groups with or without rehabilitation services. The red line is the group that did not receive rehabilitation services, and the blue line is the group that received rehabilitation services. The lower part of the axis is the logit of propensity score, and the left side of the axis is the cumulative percent

*Note* n = unweighted, %=weighted; KRW = Korean won; *p < .05, **p < .01, ***p < .001;

Figure 1 shows the standardized mean difference before and after propensity score matching. The variables used for matching were gender, age, monthly household income, degree of disability, and disability origin. The red diamond represents the standardized mean difference of the variable before matching, and the green circle represents the standardized mean difference of the variable after matching. Compared with before matching, the average mean difference of all variables after matching was close to zero. Figure 2 shows the cumulative distribution of the logit of propensity score between the with rehabilitation services group and the without rehabilitation services group. The red line represents the group without rehabilitation services group, and the blue line represents the with rehabilitation services group. The distribution between the two groups was almost identical after matching the cumulative distribution of propensity scores (Fig. 2).

### Differences in characteristics between working and non-working individuals with physical disabilities

A chi-square test and independent t-test were conducted to identify if there was a difference between working and non-working individuals with physical disabilities according to general characteristics (Table 2). There was a difference in employment according to the participants’ gender (χ^2^ = 69.158, *p* < .001), age (t = 12.996, *p* = < 0.001), education (χ^2^ = 77.699, *p* < .001), monthly household income (t=-11.095, *p* < .0010), spouse (χ^2^ = 35.044, *p* < .001), degree of disability (χ^2^ = 16.554, *p* = < 0.001), disability origin (χ^2^ = 11.175, *p* = .004), and subjective health status (t=-9.743, *p* < .001).

**Table 2:** Differences in characteristics between working and non-working individuals with physical disabilities

Variables	Categories or range	Working	Non-working	χ^2^ or t	*p*
		*n* (%) or Mean$\pm$SD			
Gender	Men	184 (65.9)	263 (36.2)	69.158	< 0.001
	Women	86 (34.1)	423 (63.8)		
Age (years)		60.58±9.99	70.21±11.09	12.996	< 0.001
Education	Elementary school	74 (27.6)	369 (52.7)	77.699	< 0.001
	Middle school	47 (19.4)	133 (20.1)		
	High school	94 (34.2)	132 (19.6)		
	$\ge$College	55 (18.8)	52 (7.6)		
Monthly household income (10,000 KRW)		269.15±176.64	137.31±132.60	-11.095	< 0.001
Spouse	Yes	189 (71.0)	335 (47.9)	35.044	< 0.001
	No^a^	81 (29.0)	351 (52.1)		
Degree of disability	Mild (grade 4–6)	220 (89.6)	469 (82.9)	16.554	< 0.001
	Severe (grade 1–3)	50 (10.4)	217 (17.1)		
Disability origin	Congenital	83 (32.9)	273 (41.6)	11.175	0.004
	Acquired	176 (62.5)	367 (51.3)		
	Unknown	11 (4.7)	46 (7.1)		
Subjective health status^b^		2.80$\pm$0.78	2.24 $\pm$0.80	-9.743	< 0.001

*Note* %=weighted; KRW = Korean won; SD = standard deviation; ^a^No spouse = widowed, divorced, separated, never married; ^b^higher scores indicate better subjective health status

### Effect of the use of rehabilitation services on the employment of persons with physical disabilities

Table 3 presents the results of the logistic regression to estimate the effect of rehabilitation services on the employment of individuals with physical disabilities. In the main analysis, there was no difference between the with rehabilitation services group and the without rehabilitation services group in employment of individuals with physical disabilities. Although not statistically significant, the utilization of rehabilitation services tended to have a positive effect on employment compared to non-utilization of rehabilitation services (OR = 1.02, *p* > .05). The subgroup analysis revealed that when the group of individuals with physical disabilities who rated their subjective health status low received rehabilitation services, it had a positive effect on employment (OR = 1.85, *p* = .018).

**Table 3:** Effect of the use of rehabilitation services on the employment of persons with physical disabilities

I. Main analysis								
Variables	Categories	B	SE	OR	95% CI		*P*	
					Lower	Higher		
Constant		-9.25	.40	.39	.32	.48	< .001	
Rehabilitation services	Yes (ref. No)	0.14	0.15	1.02	0.77	1.35	.886	

II. Subgroup analysis								
Subgroup	Categories	Rehabilitation services	B	SE	OR	95% CI		P
						Lower	Higher	
Gender	Man^1^	Yes (ref. No)	0.65	0.25	1.15	0.75	1.78	0.513
	Women^2^	Yes (ref. No)	0.62	0.29	1.17	0.72	1.90	0.536
Degree of disability	Severe^3^	Yes (ref. No)	-0.90	0.26	1.72	0.35	1.47	0.370
	Mild^4^	Yes (ref. No)	0.71	0.20	1.13	0.80	1.61	0.477
Subjective health status	Good^5^	Yes (ref. No)	-1.42	0.16	0.73	0.47	1.13	0.154
	Bad^6^	Yes (ref. No)	2.37	0.48	1.85	1.11	3.09	0.018

Log pseudo-likelihood = -569.03026; Wald chi2 = 0.02; Prob > chi2 = 0.886; Observations = 956

*Note* SE = standard error; OR = odds ratio; CI = confidence interval; ref.=reference.

1 = Log pseudolikelihood = -234.28305; Wald chi2 = 0.43; Prob > chi2 = 0.514; Observations = 344

2 = Log pseudolikelihood = -208.87204; Wald chi2 = 0.38; Prob > chi2 = 0.536; Observations = 450

3 = Log pseudolikelihood = -99.250943; Wald chi2 = 0.80; Prob > chi2 = 0.370; Observations = 220

4 = Log pseudolikelihood = -362.75179; Wald chi2 = 0.51; Prob > chi2 = 0.477; Observations = 574

5 = Log pseudolikelihood = -229.31592; Wald chi2 = 2.03; Prob > chi2 = 0.154; Observations = 334

6 = Log pseudolikelihood = -201.29251; Wald chi2 = 5.64; Prob > chi2 = 0.018; Observations = 458

## Discussion

This study aimed to investigate the impact of utilizing rehabilitation services on employment for individuals with physical disabilities aged 20 and older, using data from the 2020 National Survey of Disabled Persons. Factors that influence rehabilitation services such as gender, age, monthly household income, degree of disability, and disability origin were adjusted using propensity score matching to minimize bias between the with rehabilitation services group and without rehabilitation services group. There was no difference between the two groups in employment of individuals with physical disabilities. Compared to non-utilization, individuals with physical disabilities who rated their subjective health status low utilized rehabilitation services, it had a positive effect on employment.

The low utilization rate of rehabilitation services could be a reason their use did not affect the employment of individuals with physical disabilities in the current study. According to the 2020 National Survey of Disabled Persons, only 28.4% of individuals with physical disabilities had utilized rehabilitation services, which may have influenced our analysis results. Rehabilitation services are crucial for individuals with disabilities, as these services enable daily living activities and work. The scope of rehabilitation services for individuals with disabilities is generally limited to physical therapy and occupational therapy to enhance their health status. Moreover, rehabilitation services have tended to concentrate on specific types of disabilities, such as developmental disabilities. This concentration on certain disabilities or type of service implies that there are qualitative limitations in rehabilitation services for people with disabilities. These limitations were revealed in the 2020 National Survey of Disabled Persons. The primary reason for not utilizing rehabilitation services was, “We do not need rehabilitation services,” followed by “[we] do not know the effect” and “cost burden.” [3].

Several past studies have found that utilizing rehabilitation services did not have a significant effect on employment, suggesting that rehabilitation services are currently not directly connected to employment [19] and predominantly cater to individuals with developmental disabilities, resulting in a lack of services for those with physical disabilities. Thus, more rehabilitation services need to be established that are directly linked to employment and that gradually expand the scope of disabilities [20].

Furthermore, rather than providing rehabilitation services without an aim, it is crucial to expand the infrastructure and systems that can offer suitable services for people with physical disabilities at the national level. Similar to the Job Accommodation Network’s Searchable Online Accommodation Resource system, which enables individuals with disabilities to explore various accommodations and education online, South Korea needs to establish a national system operated by the US government that can gather and utilize information based on users’ needs [20]. Additionally, most rehabilitation services, which are currently operated by institutions, are one-off and insufficient for cultivating the occupational skills required by the labor market. Therefore, to promote employment for individuals with disabilities, it is necessary to develop customized rehabilitation services based on a job analysis of community businesses and to provide ongoing services [21].

The subgroup analysis showed that the lower subjective health status group positively affected employment when this group received rehabilitation services. However, the subgroups based on the degree of disability and gender did not significantly influence the employment of people with physical disabilities. According to the 2020 National Survey of Disabled Persons, the most common reason for not currently working was “I do not think I can do my job properly due to my health condition” [3]. These results suggest that the subjective health status of people with physical disabilities is a crucial factor in employment. Subjective health status, a subjective indicator, influenced employment, but the degree of one’s disability, an objective indicator, did not affect employment. The findings of this study align with previous research demonstrating that an individual’s subjective health assessment is a factor in work motivation [22].

Based on these findings, several recommendations can be made for rehabilitation services for people with physical disabilities. First, there is a need to consider how to increase the utilization rate of current rehabilitation services for individuals with disabilities. In particular, the necessity and effectiveness of rehabilitation services should be promoted. Second, rehabilitation services should directly impact the employment of people with physical disabilities. Third, there is a need to gradually expand the scope of rehabilitation services from services focusing on the developmentally disabled to include services for individuals with physical disabilities. Fourth, there is a need for additional education and programs aimed at improving the subjective health status of people with physical disabilities.

The present study has several limitations. First, due to the use of secondary data, we were unable to include various variables that could potentially impact the research results. For example, the quality of rehabilitation services may differ based on the content, duration, and the number of participants, all of which could affect the outcomes. Socio-cultural issues such as societal attitudes, cultural perceptions, and workplace inclusiveness initiatives also impact the employment of individuals with disabilities. Future studies should incorporate a wider range of variables influencing rehabilitation services and consider socio-cultural issues in the employment of individuals with disabilities. Second, establishing causal relationships was challenging as this study utilized cross-sectional data. As the National Survey of Disabled Persons is conducted every three years, it will be necessary to re-evaluate the findings through a longitudinal study in the future.

Despite these limitations, this study is meaningful as it focuses on the employment of individuals with disabilities. It examines whether the use of rehabilitation services affects the employment of people with disabilities by applying the propensity score matching method. This study offers insights into the direction of rehabilitation services for the future employment of this population. Furthermore, using large-scale representative sample data of individuals with disabilities in South Korea increases the potential for generalization of the research findings. Sample bias was also reduced through the use of propensity score matching.

## Conclusions

The present study identified no overall significant effect of rehabilitation services on the employment of individuals with physical disabilities. However, when individuals with physical disabilities who rated their subjective health status low received rehabilitation services, it had a positive effect on employment. The findings of this study suggest the need to review current rehabilitation services and make changes to directly impact employment. Additionally, education and programs to improve the subjective health status of people with disabilities can help enhance employment opportunities. The findings of this study can serve as foundational data for future policymaking and educational initiatives related to rehabilitation services that facilitate the employment of persons with disabilities.

### Electronic supplementary material

Below is the link to the electronic supplementary material.

Supplementary Material 1

Supplementary Material 2

## Data Availability

The datasets generated during and/or analyzed during the current study are available from the corresponding author upon reasonable request.

## Abbreviations

OR: Odds ratio
