# Supplementary material for: Impact of rehabilitation services on employment outcomes for individuals with physical disabilities: a propensity score matching analysis
Source: BMC Public Health. 2024 Jun 7;24:1534. doi: 10.1186/s12889-024-19015-6 (PMC11157936; doi:10.1186/s12889-024-19015-6)
Supplement: Supplementary file 1 — Supplementary Material 1 [file 12889_2024_19015_MOESM1_ESM.docx]

**Appendix A. Flow diagram of participants**

Missing values

(n=13)

Included in the current study

(n=1,757)

Participants aged 20 or over

(n=1,770)

Younger than 19 years

(n=3)

Other types of disabilities

(n=5,252)

Participants who have physical disabilities

(n=1,773)

Total participants in the Baseline survey

(n=7,025)
