# Supplementary material for: Impact of rehabilitation services on employment outcomes for individuals with physical disabilities: a propensity score matching analysis
Source: BMC Public Health. 2024 Jun 7;24:1534. doi: 10.1186/s12889-024-19015-6 (PMC11157936; doi:10.1186/s12889-024-19015-6)
Supplement: Supplementary file 2 — Supplementary Material 2 [file 12889_2024_19015_MOESM2_ESM.docx]

**Appendix B. Variable description used in Propensity Score Matching**

| **Variable Type** | **Variable Name** | **Description** |
| --- | --- | --- |
| Dependent  variable | Rehabilitation services | rehab_y: had experience of rehabilitation services=1, had not experience of rehabilitation services=0  rehab_n: had experience of rehabilitation services=0, had not experience of rehabilitation services=1 |
| Independent  variable | Gender | gender_m: Male = 1, Female = 0  gender_f: Male = 0, Female = 1 |
|  | Age | age_20: 20-39 = 1, others = 0  age_40: 40-49 = 1, others = 0  age_50: 50-59 = 1, others = 0  age_60: 60-69 = 1, others = 0  age_70: 70-79 = 1, others = 0  age_80: above 80 = 1, others = 0 |
|  | Monthly household income  (10,000 Korean Won) | Q1: less than 100 = 1, others = 0  Q2: above 100 ~ less than 200 = 1, others = 0  Q3: above 200 ~ less than 300 = 1, others = 0  Q4: above 300 ~ less than 400 = 1, others = 0  Q5: above 400 = 1, others = 0 |
|  | Degree of disability | drank_severe: severe = 1, mild = 0  drank_mild: severe = 0, mild = 1 |
|  | Disability origin | dorigin_pre: Congenital = 1, others = 0  dorigin_af: Acquired = 1, others = 0  dorigin_unknow: Unkown = 1, others = 0 |
